# Assessing the impact of including variation in general population mortality on standard errors of relative survival and loss in life expectancy

**DOI:** 10.1186/s12874-022-01597-7

**Published:** 2022-05-02

**Authors:** Yuliya Leontyeva, Hannah Bower, Oskar Gauffin, Paul C Lambert, Therese M.-L. Andersson

**Affiliations:** 1 Department of Medical Epidemiology and Biostatistics, Karolinska Institutet, Stockholm, Sweden; 2 Clinical Epidemiology Division, Department of Medicine Solna, Karolinska Institutet, Stockholm, Sweden; 3 Uppsala Monitoring Centre, Uppsala, Sweden; 4 Department of Health Science, Biostatistics research group, University of Leicester, Leicester, UK

**Keywords:** Relative survival, Loss in life expectancy, Flexible parametric survival models

## Abstract

**Background:**

A relative survival approach is often used in population-based cancer studies, where other cause (or expected) mortality is assumed to be the same as the mortality in the general population, given a specific covariate pattern. The population mortality is assumed to be known (fixed), i.e. measured without uncertainty. This could have implications for the estimated standard errors (SE) of any measures obtained within a relative survival framework, such as relative survival (RS) ratios and the loss in life expectancy (LLE). We evaluated the existing approach to estimate SE of RS and the LLE in comparison to if uncertainty in the population mortality was taken into account.

**Methods:**

The uncertainty from the population mortality was incorporated using parametric bootstrap approach. The analysis was performed with different levels of stratification and sizes of the general population used for creating expected mortality rates. Using these expected mortality rates, SEs of 5-year RS and the LLE for colon cancer patients in Sweden were estimated.

**Results:**

Ignoring uncertainty in the general population mortality rates had negligible (less than 1%) impact on the SEs of 5-year RS and LLE, when the expected mortality rates were based on the whole general population, i.e. all people living in a country or region. However, the smaller population used for creating the expected mortality rates, the larger impact. For a general population reduced to 0.05% of the original size and stratified by age, sex, year and region, the relative precision for 5-year RS was 41% for males diagnosed at age 85. For the LLE the impact was more substantial with a relative precision of 1286%. The relative precision for marginal estimates of 5-year RS was 3% and 30% and for the LLE 22% and 313% when the general population was reduced to 0.5% and 0.05% of the original size, respectively.

**Conclusions:**

When the general population mortality rates are based on the whole population, the uncertainty in the estimates of the expected measures can be ignored. However, when based on a smaller population, this uncertainty should be taken into account, otherwise SEs may be too small, particularly for marginal values, and, therefore, confidence intervals too narrow.

**Supplementary Information:**

The online version contains supplementary material available at (10.1186/s12874-022-01597-7).

## Background

To summarise cancer survival data various measures can be used. Within population-based studies, most of these measures are estimated in a relative survival framework, where the sometimes inaccurate or unreliable information on cause of death is not required [1]. Here, the observed mortality rate of cancer patients theoretically consists of two components: the expected mortality rate and the excess mortality rate. The excess mortality rate represents the mortality rate due to the cancer of interest and the expected mortality rate is the mortality rate due to other causes. Relative survival (RS) ratios, which are the survival analogue of excess hazards, are commonly reported at a specific time after diagnosis, usually 1-year, 5-year or 10-year relative survival. Under some assumptions RS can be interpreted as net survival [2, 3] i.e. the probability to survive if the cancer of interest was the only possible cause of death, and is useful for comparisons between groups where mortality rates due to other causes can vary. However, alternative measures that are interpreted in the presence of other causes of death are also useful. One such measure is the loss in life expectancy (LLE). The LLE is the difference in the life expectancy the cancer patients would have if they did not have cancer, and the life expectancy of the cancer population. The former life expectancy is usually assumed to be the same as the life expectancy in the general population (matched on factors like age, sex and calendar year). In comparison to RS, the LLE is defined in the "real world" since it takes into account the presence of other causes of death [4]. To estimate the LLE, the observed survival function often has to be extrapolated beyond available follow-up. It has been shown that the extrapolation performs better by extrapolating the expected and relative survival functions separately and using the interrelationship between observed, expected, and relative survival [4]. The LLE is therefore often estimated within a relative survival framework.

In practice, the expected mortality rates are usually obtained from population life tables stratified by some sociodemographic factors (such as age, sex, calendar year) and are considered known or fixed, i.e. measured without uncertainty. The argument behind this is that since the rates are based on the whole population, any uncertainty in the estimates is assumed negligible, especially in relation to the uncertainty from a considerably smaller cancer cohort. However, the mortality rates in the general population can be seen as one possible realization of the mortality rates. Even though one study showed fixed expected mortality rates to be a valid assumption for the estimation of RS [5], it might not be the case if the life tables are stratified on many variables or are based on small regions. Also, there might be situations where life tables are not available, but can be constructed from a random sample from the general population. When estimating the LLE, incorporation of uncertainty of expected mortality rates might be more important, since the expected mortality rates are included in several parts of the estimation, namely, the estimation of life expectancy in the general population and life expectancy of the cancer patients, which in turn, is estimated using the expected mortality rates and excess mortality rates.

The aim of the study was to evaluate the existing approach of estimating standard errors (SE) of RS and the LLE in comparison to if uncertainty in the expected mortality is taken into account. This is illustrated using data on colon cancer in Sweden via estimation of both marginal and conditional measures of the 5-year RS and the LLE. We use a parametric bootstrap approach to incorporate the uncertainty from the expected mortality. To investigate possible drivers of differences, we perform the analysis with different levels of stratification and sizes of the general population used for creating expected mortality rates.

## Material & methods

### Background

#### Relative survival

The mortality rate among cancer patients can be separated into two parts, the mortality rate due to the cancer of interest and the mortality rate due to other causes. In a relative survival framework where the information about the cause of death is not required, mortality due to the cancer of interest is estimated as the excess mortality among the cancer patients compared to the expected mortality in the absence of cancer. The expected mortality is based on the mortality in the general population, and it is assumed that the other-cause mortality among the cancer patients is the same as the general population mortality, matched on age, sex, calendar year and possibly other covariates. Thus, the excess mortality among cancer patients *λ*(*t*|*Z*_1_) can be written as:

1 $$ \lambda(t|Z_{1}) = h(t|Z) - h^{*}(t|Z_{2}),  $$

where *t* represents time since diagnosis, *h*(*t*|*Z*) is the all-cause mortality rate among the cancer patients and *h*^∗^(*t*|*Z*_2_) is the expected mortality. *Z* denotes a set of all covariates, while *Z*_1_ and *Z*_2_ present the covariates for excess and expected mortality respectively. The expected mortality rates are usually assumed to be known and obtained from available life tables.

After transforming mortality rates to the survival scale, relative survival (*RS*(*t*)) is defined as the ratio of all-cause survival (*S*(*t*)) and expected survival (*S*^∗^(*t*)):

2 $$ RS(t|Z_{1}) = \frac{S(t|Z)}{S^{*}(t|Z_{2})}  $$

RS is a common summary measure of cancer patients’ survival presented by national cancer registries, and is often interpreted as net survival. For RS to be interpreted as net survival, i.e. survival from cancer if there were no other possible causes of death, the assumption of exchangeability between the general population and cancer cohort must hold, i.e. the mortality in the general population must be the same as the mortality the cancer patients would have had if they did not have cancer. The other assumption is conditional independence, i.e. all the factors affecting both the cancer-specific and other-cause mortality must be controlled for [2, 3]. RS can be estimated using several approaches, both non-parametric [1, 6] and parametric [7, 8]. In this work we chose a flexible parametric survival model (FPM) within a relative survival framework [9] to model the log cumulative excess hazard. The log cumulative excess hazard within a FPM is expressed as:

3 $$ \ln[\Lambda(t|Z_{1})] = s(\ln(t)|\gamma,k_{0}) + \beta Z_{1},  $$

where *Λ*(*t*|*Z*_1_) is the cumulative excess hazard, *s*(ln(*t*)|*γ*,*k*_0_) is a restricted cubic spline function of ln(*t*) used to estimate the baseline log cumulative excess hazard [10]. The model (3) is a proportional excess hazards model but it can be easily extended to non proportional hazards by incorporating time dependent effects. This can be done by forming interactions between the covariates of interest and the spline terms for time [9].

Based on model (3) and the general relationship between the cumulative hazard function and the survival function, *RS*(*t*) can be obtained by

4 $$ RS(t|Z_{1})=\exp(-\exp(\ln[\Lambda(t|Z_{1})])).  $$

#### The loss in life expectancy

The loss in life expectancy (LLE) is the difference between life expectancy in the general population, free from the cancer of interest, *LE*_*P*_, and the life expectancy in the cancer population, *LE*_*C*_:

$$LLE(Z) = LE_{P}(Z_{2}) - LE_{C}(Z), $$

*LE*_*C*_ can be calculated as the area under all-cause survival curve *S*(*t*):

$$LE_{C}(Z) = \int_{0}^{\infty}S(u|Z)du. $$

Similarly, *LE*_*P*_ equals the area under general population survival or expected survival *S*^∗^(*t*):

$$LE_{P}(Z_{2}) = \int_{0}^{\infty}S^{*}(u|Z_{2})du $$

Thus, LLE can be written as:

5 $$  LLE(Z) = \int_{0}^{\infty}S^{*}(u|Z_{2})du - \int_{0}^{\infty}S(u|Z)du  $$

Assuming that the cancer patients would have had the same life expectancy as the general population, had they not been diagnosed with cancer, LLE estimates the number of years the life expectancy is reduced due to cancer. Theoretically, LLE is easy to estimate (Eq. (5)), however, in practice the estimation often requires extrapolation of the survival functions due to limited follow-up. It has been shown that extrapolation of the all-cause survival curve *S*(*t*) is preferably performed by breaking it into two components: relative survival *RS*(*t*) and expected survival *S*^∗^(*t*) [4], and extrapolating the functions separately. As a result, LLE is estimated by:

6 $$ LLE(Z) \!= \int_{0}^{\infty}S^{*}(u|Z_{2})du - \int_{0}^{\infty}RS(u|Z_{1})S^{*}(u|Z_{2})du,  $$

where expected survival *S*^∗^(*t*) is obtained using population life tables and *RS*(*t*) is obtained from a FPM (Eq. (4)),

7 $$ \begin{aligned} LLE(Z) =& \int_{0}^{\infty}S^{*}(u|Z_{2})du - \int_{0}^{\infty}\\ &\exp(-\exp(\ln[\Lambda(t|Z_{1})]))S^{*}(u|Z_{2})du. \end{aligned}  $$

#### Marginal measures

For population-based cancer survival, interest often lies in obtaining an average estimate (a single number) for RS or LLE, across the covariate distribution. In other words, we are interested in marginal estimands, which can be estimated using regression standardization [11].

Marginal relative survival (*RS*_*m*_(*t*)) is defined as the expectation over the distribution of covariates *Z*_1_ and can be estimated by predicting relative survival for all individuals in the cancer population at time *t* after diagnosis and averaging them [12]:

$$\widehat{RS}_{m}(t) = \frac{1}{N}\sum_{i = 1}^{N}\widehat{RS}_{i}(t|Z_{1i}), $$

where $\widehat {RS}_{i}(t|Z_{1i})$ is the predicted RS for individual *i* at time *t*, *Z*_1*i*_ is the covariate pattern for individual *i* associated with the excess mortality, and *N* is the number of all individuals in the cancer population.

Analogically to marginal RS, marginal loss in expectation of life (*LLE*_*m*_) is estimated by taking the average over the predicted LLE of all individuals in the data set:

$$\widehat{LLE}_{m} = \frac{1}{N}\sum_{i=1}^{N} \widehat{LLE}_{i}(Z_{i}) $$

#### Variance estimation

The model parameters in Eq. (3) are obtained using maximum likelihood, assuming that the expected mortality is fixed (i.e. measured without uncertainty). Therefore, the variance in the estimation of the log cumulative excess hazard is based solely on the cancer cohort data. RS is estimated from the model as shown in Eq. (4), and the SE of RS is obtained using the delta method. Confidence intervals (CI) of RS are first obtained on the log cumulative excess hazard scale (the scale we are modelling on), and then transformed to the survival scale (using Eq. (4)). The variance of LLE is also based on the delta method, where the uncertainty solely comes from the estimation of excess mortality. Therefore, the assumption that the expected mortality is measured without uncertainty is used three times for LLE. First in the estimation of *RS*(*t*) using a FPM, then when multiplying *RS*(*t*) with the expected survival *S*^∗^(*t*) when obtain the life expectancy among the cancer patients *LE*_*C*_, and lastly by taking the life expectancy among the general population *LE*_*P*_ as a constant. The delta method is used to obtain the variance of marginal measures as well.

#### Population mortality rates

The general population mortality used for the expected mortality rate *h*^∗^(*t*) and the expected survival *S*^∗^(*t*) for the estimation of RS and LLE are often obtained from statistics bureaus and presented on a national level. In other words, the estimates of the general population mortality are based on the whole population, i.e. all people living in a country or region, that is the catchment area for the population-based cancer registry. In this study the population mortality rates are based on all people living in Sweden. The fact that *h*^∗^(*t*) and *S*^∗^(*t*) are based on the whole population is the reason why they are assumed fixed, and measured without uncertainty. However, this assumption might be questioned because *h*^∗^(*t*) and *S*^∗^(*t*) can be seen as one potential realization from a random process. Also, there might be scenarios when uncertainty from the expected mortality rates should be taken into account. For instance, when one wants to use population mortality rates stratified by many covariates. Often, the population mortality rates are stratified by age, sex and calendar year. However, the expected mortality can also differ across regions or for various socioeconomic status. Then, the population mortality rates will also be stratified by region, socioeconomic status or other covariates. Consequently, when many stratified variables are employed, the number of people in each stratified cell can be very small and thus, ignoring uncertainty in the expected mortality rates might be inaccurate. There are, in addition, scenarios when one would like to stratify the expected mortality rates by factors which are not available on a national level. If the data for the whole population are not available, expected rates can be constructed based on a random sample of individuals from the whole population, where information on the missing variables is available [13].

### Material

#### Cohort data

Sweden has a population size of approximately 10,000,000, and all cancer cases are reported to the Cancer Register. In this study, we used data from the Swedish Cancer Registry to identify patients diagnosed with colon cancer in Sweden in 2006. Only cases aged 50 and older at diagnosis were included, and cases diagnosed at autopsy were excluded. In total, 3400 patients were included in this study. The patients were followed from diagnosis to death due to any cause or the end of 2017, whichever came first. A 10% random sample from the cancer cohort (318 patients) was also created to be able to investigate how the estimates could be affected in a smaller population, for example a smaller country or region.

This study was approved by the Swedish Ethical Review Authority. Informed consent from study subjects was not required for the current study. This study was carried out in accordance with the Declaration of Helsinki, and all methods were carried out in accordance with relevant guidelines and regulations in Sweden.

#### General population data

We used two data sets that contained the number of deaths and person-years in the Swedish population, obtained from Statistics Sweden [14]. The first data set was stratified by sex, yearly age from 18 to 99 and calendar year from 1975 to 2017. We denote it *popmort*. The second dataset was stratified by an additional factor, region, and we denote it *popmort_region*. Sweden is divided into 21 regions, the largest being Stockholm with a population size of approximately 2,300,000 and the smallest is Gotland with a population size of approximately 60,000. Both *popmort* and *popmort_region* are based on the whole general population, i.e. all people living in Sweden. We refer to them as population mortality files with original size. As mentioned above, there are scenarios when the expected rates might not be based on the whole population. To address this, we created population mortality files based on the datasets obtained from Statistics Sweden, but reduced in size. To do this *popmort* and *popmort_region* were reduced in size by a factor of 10, 200 and 2000 (both the number of deaths and person-years were divided by 10, 200 and 2000). Thus, we obtained population mortality files with a population of 1 million, 50,000 and 5,000 people, which corresponds to 10%, 0.5% and 0.05% of the original size. This gave us in total 8 different versions of general population mortality data.

#### Underlying and varying population mortality rates

To compare the standard errors of RS and LLE estimated with the existing approach, and an approach which takes the uncertainty in the general population mortality into account, we need both fixed, or *underlying*, population mortality rates and *varying* population mortality rates. To obtain these mortality rates we fitted Poisson regression models to the two original data sets of population death counts described above. The models included the covariates sex, age and year with the log person-years as an offset. Age and year were treated as continuous variables modeled using restricted cubic splines. In addition, the pairwise interaction terms of age, gender and year were included into the model to allow for differential effects across groups. For the general population mortality including region, separate models were fitted to each region. The predictions from the Poisson models were used as underlying expected mortality rates. Thus, two obtained files were used as data sets with underlying expected mortality rates in the analysis, with and without stratification by region. The reason why the underlying mortality rates were constructed from modeling, instead of directly using the data for each covariate pattern, was to make them comparable to the varying mortality rates. However, the modelled rates were close to the original mortality rates obtained from Statistics Sweden. To be able to get varying expected mortality rates, bootstrapping from the models described were used, as outlined below.

To incorporate uncertainty of the expected mortality in the estimation of the standard errors of the 5-year RS and LLE we created a set of 1000 realizations of the expected mortality rates and expected survival probabilities. To do this, we used a parametric bootstrap. For each of the 8 different versions of the general population mortality data abovementioned (with a different size of the source population and with / without stratification by region) we fitted the Poisson model described above. Since smaller general populations were created by dividing the number of deaths and person-years by a corresponding factor, we obtained the same $\widehat {\beta }$ coefficients but different variance-covariance matrices $\widehat {\Sigma }$ from the Poisson model in all underlying population sizes. New *β* parameters were drawn 1000 times from a multivariate normal distribution using the vector of $\widehat {\beta }$ coefficients and the variance-covariance matrix $\widehat {\Sigma }, N(\widehat \beta, \widehat \Sigma)$ from the Poisson model [15]. For each draw, the expected mortality rates were obtained from the generated *β* parameters. These 1000 imputed data sets were used as the replicates of the underlying expected mortality rates, resulting in *popmort*_1_- *popmort*_1000_ varying expected mortality rates for each of 8 population mortality files (with / without region and 4 sizes of the population).

### Methods

#### Conventional estimates

To obtain conventional estimates, namely, the estimates with the approach assuming no uncertainty in the expected mortality rates, FPMs within a relative survival framework (as shown in Eq. (3)) were fitted. We investigated 4 different settings. For settings 1 and 2, the full cancer cohort and mortality rates based on the general population of original size were used. For setting 1 population mortality rates were stratified by age, year and sex, while for setting 2 population mortality rates were stratified by age, year, sex and region. Within settings 1S and 2S the cancer cohort that was reduced to 10% of the original size was used to investigate what would be observed in a smaller population (here S stands for small). In these settings, the population mortality rates based on the population reduced to 10% of the original size were used. Similarly to settings 1 and 2, for setting 1S the population mortality rates were stratified by age, year and sex, and for setting 2S also by region.

To obtain the conventional estimates for each of these settings, 4 FPMs were fitted. Age at diagnosis and sex were included in the models, and the time-scale being time from diagnosis. Region was not included into the model, i.e. it was assumed that the excess mortality is the same in all regions. The expected mortality, however, depends on region if the underlying expected mortality rates are stratified by region and does not, otherwise. The log baseline cumulative excess hazard was estimated using restricted cubic splines with 5 degrees of freedom (df). Age was included as a continuous variable using restricted cubic splines with 4 df. Furthermore, restricted cubic splines with 3 df were used to capture the time-varying effect of age and sex. Within all fitted models, the expected mortality rates were assumed to be fixed.

Based on these 4 FPMs, the 5-year RS, LLE and their SEs were estimated for each of the 4 settings of interest. In the estimation of LLE, we used the same population mortality file as used in the modelling step, again assuming known expected mortality rates, i.e. by using the underlying expected mortality rates as described above. 5-year RS by age and sex, as well as marginal 5-year RS were estimated. Since the LLE depends on not only the excess mortality but also the life expectancy in the general population, it will also vary by the factors that the expected mortality rates are stratified by. Thus, with region-specific expected mortality rates the LLE was obtained by age, sex and region. Marginal LLE was also estimated.

#### Variance estimation including uncertainty in the expected mortality

To estimate the SEs of the 5-year RS and LLE when the variation in the expected mortality is taken into account, the FPMs described above were also modeled using the 1000 varying expected population mortality rates obtained with parametric bootstrap. For each of the above-mentioned settings 1 and 2 we investigated 4 scenarios using different sizes of the general population as described previously. For the settings 1S and 2S only one scenario each was employed. This gave in total 10 different scenarios that were studied, and these are summarized in Table 1. Therefore, for each scenario from Table 1 we fitted 1000 FPMs (using each of the 1000 varying expected mortality rates) and obtained the 5-year RS, LLE and their SEs estimates for each and every model in the same way as the conventional estimates.

**Table 1:** Outline of different scenarios to incorporate uncertainty in the expected mortality

Scenario	cancer cohort used	size of general population used for population mortality	population mortality stratified by	corresponding conventional setting
A	full	original	age, year, sex	1
B	full	reduced to 10%	age, year, sex	1
C	full	reduced to 0.5%	age, year, sex	1
D	full	reduced to 0.05%	age, year, sex	1
E	reduced to 10%	reduced to 10%	age, year, sex	1S^a^
F	full	original	age, year, sex, region	2
G	full	reduced to 10%	age, year, sex, region	2
H	full	reduced to 0.5%	age, year, sex, region	2
I	full	reduced to 0.05%	age, year, sex, region	2
J	reduced to 10%	reduced to 10%	age, year, sex, region	2S^a^

^a^S stands for small and refers to the setting to investigate what would be observed for a smaller population

As described above, to include the uncertainty of the expected mortality rates in the estimates of RS and LLE, for each of 10 scenarios from Table 1, we fitted the FPMs 1000 times, using each of 1000 replicates of the underlying expected mortality rates for that specific scenario. Each time, the conditional 5-year RS and LLE, and marginal RS and LLE, and their SEs were obtained. Finally, using Rubin’s rules [16] the estimates were combined to derive the pooled estimates and standard errors.

For estimates of LLE, the pooled mean was estimated as:

$$\overline{LLE}_{p} = \frac {\sum_{i = 1}^{M}\widehat{LLE}_{i}}{M}, $$

and the pooled variance as

$$V_{p} = V_{W} + V_{B} + \frac{V_{B}}{M}, $$

where *V*_*W*_ is within imputation variance, *V*_*B*_ is between imputation variance and *M* is the number of the imputed data sets.

$$V_{W} = \frac{1}{M}\sum_{i = 1}^{M}\widehat {SE}_{i}^{2}, $$

where $\widehat {SE}_{i}$ is a standard error for $\widehat {LLE}_{i}, i= 1,..., M$

$$V_{B} = \frac{\sum_{i = 1}^{M}(\widehat {LLE}_{i} - {\overline{LLE}_{p}})^{2}}{M-1} $$

The above equations show the marginal estimates, however, the same approach was used for conditional estimates. The estimates for 5-year RS were obtained in the same way. These estimates are denoted by estimates obtained with a bootstrap-based method.

#### Performance measure

To compare the standard errors from the bootstrap-based methods to the conventional method the relative % precision (RP) [17] was calculated by:

$$RP = 100\left(\left(\frac{\widehat{SE}_{boot}}{\widehat{SE}_{conv}}\right)^{2} - 1\right), $$

where $\widehat {SE}_{boot}$ and $\widehat {SE}_{conv}$ are estimated SEs of 5-year RS or LLE, obtained with the bootstrap-based and conventional methods, respectively.

The analysis was performed with Stata 15.1 software packages stpm2 and standsurv available publicly [8, 18, 19].

## Results

***Conventional setting 1***

The point estimates (PE) of 5-year relative survival and loss in life expectancy by selected ages at diagnosis (55, 65, 75, 85) and sex are presented in Table 2 for scenarios A-D (using population mortality rates stratified by age, sex and calendar year), as well as for the corresponding conventional setting 1. The SEs, CIs and RP for each of the estimates are also shown. It can be seen that SEs obtained with a bootstrap-based method are larger than conventional SEs for scenario D (when the size of the general population is reduced to 0.05% of the original size). For 5-year RS, this increase is noticeable for patients older than 75 years, while for LLE changes are seen for all ages. In addition, the increase is larger for LLE than for RS. For example, the relative precision of 5-year RS for scenario D for males aged 75 is approximately 15%, while the RP of LLE in the same scenario is approximately 221%.

**Table 2:** Estimates of 5-year RS and LLE for setting 1 and scenarios A-D. Point estimates (PE) of 5-year relative survival (RS) and loss in life expectancy (LLE), with lower (LCI) and upper (UCI) confidence intervals, standard errors (SE) and relative % precision (RP) from setting 1, different methods and scenarios for including uncertainty in general population mortality when estimating SEs. Results are presented for men and women, aged 55, 65, 75 and 85 years at diagnosis. General population mortality rates are stratified by age, sex and calendar year. RP illustrates comparison of the conventional method to a bootstrap-based method

Method	Setting / scenario^b^	5-year Relative Survival (RS)					Loss in life expectancy (LLE)^a^				
		PE	SE	LCI	UCI	RP^c^	PE	SE	LCI	UCI	RP
*men, 55 years at diagnosis*											
Conventional^d^	1	0.638	0.026	0.585	0.687	0.00	12.57	0.86	10.90	14.25	0.00
Bootstrap	A	0.638	0.026	0.585	0.687	0.00	12.57	0.86	10.90	14.25	0.03
-based^e^	B	0.638	0.026	0.585	0.687	0.01	12.57	0.86	10.89	14.26	0.30
	C	0.638	0.026	0.585	0.687	0.14	12.58	0.88	10.85	14.31	6.22
	D	0.639	0.026	0.585	0.687	1.39	12.57	1.07	10.46	14.68	57.57
*men, 65 years at diagnosis*											
Conventional	1	0.636	0.021	0.593	0.675	0.00	7.69	0.44	6.83	8.54	0.00
Bootstrap	A	0.636	0.021	0.593	0.675	0.00	7.69	0.44	6.83	8.54	0.06
-based	B	0.636	0.021	0.593	0.675	0.03	7.69	0.44	6.83	8.54	0.60
	C	0.636	0.021	0.593	0.675	0.48	7.69	0.46	6.79	8.60	12.69
	D	0.636	0.022	0.592	0.677	4.83	7.69	0.65	6.42	8.95	120.05
*men, 75 years at diagnosis*											
Conventional	1	0.629	0.021	0.586	0.669	0.00	4.19	0.23	3.74	4.65	0.00
Bootstrap	A	0.629	0.021	0.586	0.669	0.01	4.19	0.23	3.74	4.65	0.11
-based	B	0.629	0.021	0.586	0.669	0.07	4.19	0.23	3.73	4.65	1.06
	C	0.629	0.021	0.585	0.669	1.40	4.20	0.26	3.70	4.70	22.25
	D	0.629	0.023	0.583	0.672	14.76	4.21	0.42	3.39	5.02	220.92
*men, 85 years at diagnosis*											
Conventional	1	0.585	0.030	0.523	0.641	0.00	1.90	0.13	1.64	2.16	0.00
Bootstrap	A	0.585	0.030	0.523	0.641	0.02	1.90	0.13	1.64	2.16	0.12
-based	B	0.585	0.030	0.523	0.641	0.21	1.90	0.13	1.64	2.16	1.27
	C	0.584	0.031	0.521	0.642	4.12	1.90	0.15	1.61	2.19	24.31
	D	0.584	0.036	0.511	0.651	40.37	1.92	0.25	1.43	2.40	244.55
*women, 55 years at diagnosis*											
Conventional	1	0.663	0.025	0.611	0.709	0.00	12.06	0.90	10.30	13.83	0.00
Bootstrap	A	0.663	0.025	0.611	0.709	0.00	12.06	0.90	10.30	13.83	0.02
-based	B	0.663	0.025	0.611	0.709	0.01	12.06	0.90	10.29	13.83	0.19
	C	0.663	0.025	0.611	0.709	0.34	12.06	0.92	10.26	13.86	4.23
	D	0.663	0.025	0.611	0.710	3.74	12.04	1.08	9.92	14.16	44.26
*women, 65 years at diagnosis*											
Conventional	1	0.660	0.020	0.619	0.697	0.00	7.76	0.47	6.84	8.68	0.00
Bootstrap	A	0.660	0.020	0.619	0.697	0.00	7.76	0.47	6.84	8.68	0.04
-based	B	0.660	0.020	0.619	0.697	0.02	7.76	0.47	6.84	8.69	0.36
	C	0.660	0.020	0.619	0.697	0.44	7.76	0.49	6.81	8.72	7.87
	D	0.660	0.020	0.619	0.698	4.70	7.75	0.63	6.51	8.99	80.65
*women, 75 years at diagnosis*											
Conventional	1	0.653	0.020	0.612	0.692	0.00	4.56	0.27	4.03	5.09	0.00
Bootstrap	A	0.653	0.020	0.612	0.692	0.00	4.56	0.27	4.03	5.09	0.07
-based	B	0.653	0.020	0.612	0.692	0.03	4.56	0.27	4.03	5.09	0.64
	C	0.653	0.020	0.612	0.692	0.68	4.56	0.29	4.00	5.13	13.01
	D	0.654	0.021	0.611	0.693	6.59	4.56	0.41	3.76	5.37	129.26
*women, 85 years at diagnosis*											
Conventional	1	0.611	0.027	0.556	0.662	0.00	2.20	0.15	1.90	2.50	0.00
Bootstrap	A	0.611	0.027	0.556	0.662	0.01	2.20	0.15	1.90	2.50	0.09
-based	B	0.611	0.027	0.556	0.662	0.18	2.20	0.15	1.90	2.50	0.90
	C	0.611	0.028	0.555	0.662	3.42	2.20	0.17	1.88	2.52	17.56
	D	0.611	0.031	0.547	0.669	33.92	2.22	0.25	1.72	2.71	175.91

^a^LLE is presented in years

^b^See Table 1 for information on the different settings and scenarios

^c^RP is presented in %

^d^Conventional refers to the standard method for estimating SEs, where the general population mortality is assumed to be measured without uncertainty

^e^Bootstrap-based refers to the parametric bootstrap approach used for including uncertainty in population mortality rates in the estimation of SEs

***Conventional setting 2***

Table 3 illustrates estimates for selected ages at diagnosis (55, 65, 75, 85) and by sex from scenarios F-I (using population mortality rates stratified by age, sex, calendar year and region), as well as from the corresponding conventional setting 2. Since the estimates of LLE also differ by region in these scenarios, the results are shown for the Stockholm region. Similar patterns to scenarios A-D can be seen here. The bootstrap-based SEs are larger than the conventional SEs for scenario I (when the expected mortality is based on a population reduced to 0.05% of the original size). The increase in the SEs of LLE estimates can also be observed with scenario H (the general population is reduced to 0.5% of the original size). For example, the SE of LLE for males aged 55 is 0.87 for conventional method, scenarios F and G (the general population of original size and reduced to 10% of the original size, respectively), while for scenario H (the size of the general population is 0.5% of the original size) the SE of LLE is 0.94 and 1.47 for scenario I (when the general population is 0.05% of the original size). Similar to the estimates from scenarios A-D, the increase is larger for LLE than for RS. In addition, the RP of LLE is larger than the RP of LLE in setting 1 (using population mortality rates stratified by age, sex and calendar year). For example, for men aged 75 years in setting 1, scenario D, the RP of LLE is about 221%, while in setting 2, scenario I, the RP is 811%. The same pattern is seen for the smallest region in Sweden, the Gotland region, although the RP is much higher for the Gotland region than for the Stockholm region. The results for the Gotland region can be found in Additional file 1 for scenarios F-H, and Additional file 2 for scenario J.

**Table 3:** Estimates of 5-year RS and LLE for setting 2 and scenarios F-I. Point estimates (PE) of 5-year relative survival (RS) and loss in life expectancy (LLE), with lower (LCI) and upper (UCI) confidence intervals, standard errors (SE) and relative % precision (RP) from setting 2, different methods and scenarios for including uncertainty in the general population mortality when estimating SEs. Results are presented for men and women, aged 55, 65, 75 and 85 years at diagnosis, and LLE estimates are for the Stockholm region. General population mortality rates are stratified by age, sex, calendar year and region. RP illustrates comparison of the conventional method to a bootstrap-based method

Method	Setting / scenario^b^	5-year Relative Survival (RS)					Loss in life expectancy (LLE)^a,^^f^				
		PE	SE	LCI	UCI	RP^c^	PE	SE	LCI	UCI	RP
*men, 55 years at diagnosis*											
Conventional^d^	2	0.639	0.026	0.585	0.687	0.00	12.74	0.87	11.03	14.44	0.00
Bootstrap^e^	F	0.639	0.026	0.585	0.687	0.00	12.74	0.87	11.03	14.44	0.10
-based	G	0.639	0.026	0.585	0.687	0.01	12.74	0.87	11.02	14.45	0.86
	H	0.639	0.026	0.585	0.687	0.23	12.72	0.94	10.87	14.57	17.68
	I	0.640	0.026	0.586	0.689	2.32	12.73	1.47	9.84	15.62	186.49
*men, 65 years at diagnosis*											
Conventional	2	0.636	0.021	0.593	0.675	0.00	7.83	0.45	6.95	8.70	0.00
Bootstrap	F	0.636	0.021	0.593	0.675	0.00	7.83	0.45	6.95	8.70	0.20
-based	G	0.636	0.021	0.593	0.675	0.03	7.83	0.45	6.95	8.71	1.81
	H	0.636	0.021	0.593	0.676	0.55	7.82	0.52	6.80	8.85	37.16
	I	0.638	0.022	0.594	0.679	5.58	7.89	1.00	5.92	9.85	403.74
*men, 75 years at diagnosis*											
Conventional	2	0.629	0.021	0.586	0.669	0.00	4.34	0.24	3.86	4.81	0.00
Bootstrap	F	0.629	0.021	0.586	0.669	0.01	4.34	0.24	3.86	4.81	0.37
-based	G	0.629	0.021	0.586	0.669	0.07	4.34	0.25	3.86	4.82	3.21
	H	0.629	0.021	0.586	0.670	1.43	4.34	0.31	3.73	4.95	65.54
	I	0.630	0.023	0.584	0.673	13.86	4.45	0.73	3.02	5.88	810.92
*men, 85 years at diagnosis*											
Conventional	2	0.581	0.030	0.520	0.638	0.00	2.03	0.14	1.75	2.30	0.00
Bootstrap	F	0.581	0.030	0.520	0.638	0.03	2.03	0.14	1.75	2.30	0.46
-based	G	0.581	0.030	0.519	0.638	0.20	2.03	0.14	1.74	2.31	3.96
	H	0.580	0.031	0.517	0.638	3.95	2.04	0.19	1.66	2.41	84.42
	I	0.575	0.036	0.502	0.642	41.27	2.16	0.53	1.13	3.19	1286.02
*women, 55 years at diagnosis*											
Conventional	2	0.662	0.025	0.611	0.709	0.00	12.26	0.92	10.46	14.06	0.00
Bootstrap	F	0.662	0.025	0.611	0.709	0.00	12.26	0.92	10.46	14.06	0.05
-based	G	0.662	0.025	0.611	0.709	0.01	12.26	0.92	10.45	14.06	0.48
	H	0.663	0.025	0.611	0.709	0.36	12.25	0.96	10.36	14.14	10.37
	I	0.665	0.025	0.612	0.711	4.08	12.18	1.30	9.64	14.72	99.20
*women, 65 years at diagnosis*											
Conventional	2	0.660	0.020	0.619	0.697	0.00	7.93	0.48	6.99	8.88	0.00
Bootstrap	F	0.660	0.020	0.619	0.697	0.01	7.93	0.48	6.99	8.88	0.10
-based	G	0.660	0.020	0.619	0.697	0.02	7.93	0.48	6.98	8.88	1.04
	H	0.660	0.020	0.619	0.697	0.48	7.93	0.53	6.88	8.97	21.90
	I	0.662	0.020	0.621	0.701	5.50	7.93	0.85	6.26	9.61	214.02
*women, 75 years at diagnosis*											
Conventional	2	0.653	0.020	0.612	0.692	0.00	4.73	0.28	4.18	5.29	0.00
Bootstrap	F	0.653	0.020	0.612	0.692	0.01	4.73	0.28	4.18	5.29	0.18
-based	G	0.653	0.020	0.612	0.692	0.03	4.73	0.29	4.17	5.29	1.71
	H	0.654	0.020	0.612	0.692	0.66	4.73	0.33	4.09	5.38	36.30
	I	0.655	0.021	0.612	0.695	6.68	4.80	0.64	3.55	6.05	406.77
*women, 85 years at diagnosis*											
Conventional	2	0.607	0.027	0.553	0.658	0.00	2.35	0.16	2.04	2.67	0.00
Bootstrap	F	0.608	0.027	0.553	0.658	0.03	2.35	0.16	2.03	2.67	0.25
-based	G	0.607	0.027	0.552	0.658	0.15	2.36	0.16	2.03	2.68	2.28
	H	0.607	0.027	0.551	0.658	3.13	2.37	0.20	1.98	2.75	48.53
	I	0.602	0.031	0.539	0.660	32.32	2.48	0.47	1.56	3.39	718.93

^a^LLE is presented in years

^b^See Table 1 for information on the different settings and scenarios

^c^RP is presented in %

^d^Conventional refers to the standard method for estimating SEs, where the general population mortality is assumed to be measured without uncertainty

^e^Bootstrap-used refers to the parametric bootstrap approach used for including uncertainty in population mortality rates in the estimation of SEs

^f^LLE estimates are presented for the Stockholm region

***Conventional settings 1S and 2S***

For the scenarios where the cancer cohort is reduced in size (scenarios E and J) an inflation in SEs was not observed, for either 5-year RS or LLE, regardless whether the population mortality rates were stratified by region or not (Table 4).

**Table 4:** Estimates of 5-year RS and LLE for settings 1S, 2S and scenarios E, J. Point estimates (PE) of 5-year relative survival (RS) and loss in life expectancy (LLE), with lower (LCI) and upper (UCI) confidence intervals, standard errors (SE) and relative % precision (RP) from different methods, settings and scenarios for including uncertainty in the general population mortality when estimating SEs. Results are presented for men and women, aged 55, 65, 75 and 85 years at diagnosis, and in setting 2S, scenario J the LLE estimates are for the Stockholm region. For setting 1S general population mortality rates are stratified by age, sex and calendar year. For setting 2S general population mortality rates are stratified by age, sex, calendar year and region. RP illustrates comparison of the conventional method to a bootstrap-based method

Method	Setting / scenario^b^	5-year Relative Survival (RS)					Loss in life expectancy (LLE)^a,^^g^				
		PE	SE	LCI	UCI	RP^c^	PE	SE	LCI	UCI	RP
*men, 55 years at diagnosis*											
Conventional^d^	1S^f^	0.639	0.102	0.406	0.801	0.00	11.28	3.08	5.24	17.31	0.00
Bootstrap-based^e^	E	0.639	0.102	0.405	0.801	0.01	11.28	3.08	5.24	17.31	0.03
*men, 65 years at diagnosis*											
Conventional	1S	0.670	0.072	0.507	0.790	0.00	7.47	1.50	4.53	10.41	0.00
Bootstrap-based	E	0.670	0.072	0.507	0.790	0.00	7.47	1.50	4.53	10.41	0.06
*men, 75 years at diagnosis*											
Conventional	1S	0.510	0.075	0.357	0.644	0.00	5.81	0.72	4.39	7.23	0.00
Bootstrap-based	E	0.510	0.075	0.357	0.644	0.00	5.81	0.72	4.39	7.23	0.16
*men, 85 years at diagnosis*											
Conventional	1S	0.619	0.100	0.394	0.781	0.00	1.74	0.44	0.88	2.60	0.00
Bootstrap-based	E	0.619	0.101	0.394	0.781	0.03	1.74	0.44	0.88	2.60	0.18
*women, 55 years at diagnosis*											
Conventional	1S	0.672	0.099	0.440	0.825	0.00	8.83	2.89	3.17	14.49	0.00
Bootstrap-based	E	0.672	0.099	0.440	0.825	0.00	8.83	2.89	3.17	14.49	0.00
*women, 65 years at diagnosis*											
Conventional	1S	0.701	0.060	0.566	0.801	0.00	6.49	1.47	3.61	9.37	0.00
Bootstrap-based	E	0.701	0.060	0.566	0.801	0.00	6.49	1.47	3.61	9.37	0.04
*women, 75 years at diagnosis*											
Conventional	1S	0.550	0.082	0.377	0.693	0.00	5.98	1.05	3.92	8.05	0.00
Bootstrap-based	E	0.550	0.082	0.377	0.693	0.00	5.98	1.05	3.92	8.05	0.07
*women, 85 years at diagnosis*											
Conventional	1S	0.653	0.082	0.470	0.787	0.00	1.89	0.46	0.98	2.80	0.00
Bootstrap-based	E	0.653	0.082	0.470	0.787	0.03	1.89	0.46	0.98	2.80	0.11
*men, 55 years at diagnosis*											
Conventional^d^	2S^f^	0.637	0.103	0.404	0.800	0.00	11.49	3.12	5.38	17.60	0.00
Bootstrap-based^e^	J	0.637	0.102	0.404	0.800	-0.01	11.49	3.12	5.38	17.61	0.05
*men, 65 years at diagnosis*											
Conventional	2S	0.668	0.072	0.505	0.788	0.00	7.65	1.54	4.64	10.66	0.00
Bootstrap-based	J	0.668	0.072	0.505	0.788	0.00	7.65	1.54	4.63	10.67	0.16
*men, 75 years at diagnosis*											
Conventional	2S	0.509	0.074	0.356	0.643	0.00	6.03	0.75	4.56	7.51	0.00
Bootstrap-based	J	0.509	0.074	0.356	0.643	0.00	6.03	0.76	4.55	7.51	0.62
*men, 85 years at diagnosis*											
Conventional	2S	0.612	0.100	0.389	0.774	0.00	1.88	0.47	0.96	2.79	0.00
Bootstrap-based	J	0.612	0.100	0.389	0.774	0.01	1.88	0.47	0.96	2.80	0.50
*women, 55 years at diagnosis*											
Conventional	2S	0.673	0.098	0.443	0.825	0.00	8.93	2.94	3.17	14.69	0.00
Bootstrap-based	J	0.673	0.098	0.443	0.825	-0.01	8.93	2.94	3.17	14.69	0.03
*women, 65 years at diagnosis*											
Conventional	2S	0.702	0.060	0.567	0.802	0.00	6.59	1.50	3.64	9.54	0.00
Bootstrap-based	J	0.702	0.060	0.567	0.802	0.00	6.59	1.50	3.65	9.54	0.06
*women, 75 years at diagnosis*											
Conventional	2S	0.553	0.082	0.380	0.695	0.00	6.17	1.11	4.00	8.33	0.00
Bootstrap-based	J	0.553	0.082	0.380	0.695	0.00	6.17	1.11	4.00	8.33	0.14
*women, 85 years at diagnosis*											
Conventional	2S	0.649	0.081	0.467	0.783	0.00	2.03	0.50	1.05	3.01	0.00
Bootstrap-based	J	0.649	0.081	0.467	0.783	0.00	2.03	0.50	1.05	3.01	0.20

^a^LLE is presented in years

^b^See Table 1 for information on the different settings and scenarios

^c^RP is presented in %

^d^Conventional refers to the standard method for estimating SEs, where the general population mortality is assumed to be measured without uncertainty

^e^Bootstrap-based refers to the parametric bootstrap approach used for including uncertainty in population mortality rates in the estimation of SEs

^f^S stands for small and refers to the setting to investigate what would be observed for a smaller population

^g^LLE estimates in setting 2S and scenario J are presented for the Stockholm region

### Confidence intervals

**Fig. 1:**
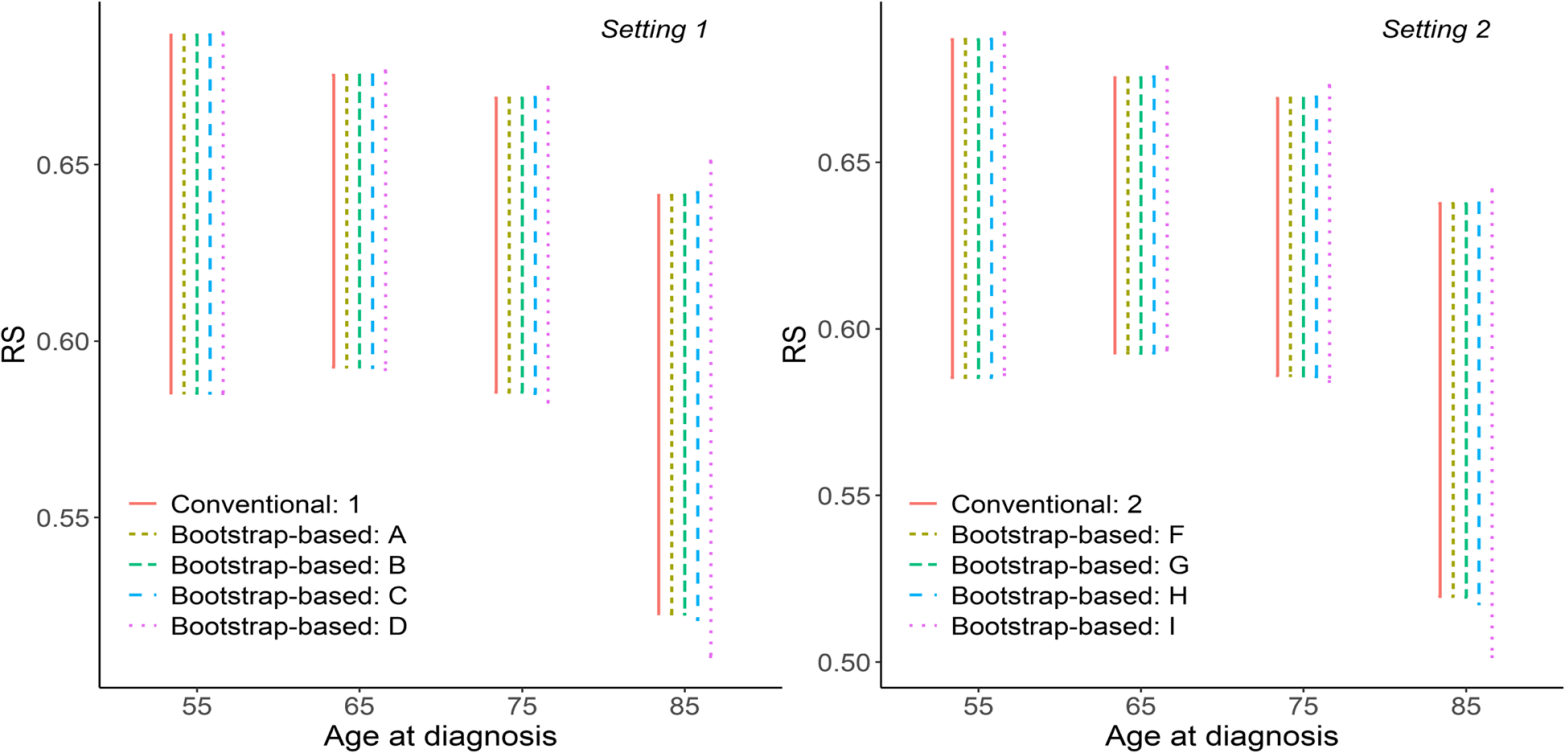
Confidence intervals of 5-year RS. Confidence intervals of 5-year relative survival (RS) from different methods, settings and scenarios for including uncertainty in the general population mortality when estimating SEs. Conventional refers to the standard method for estimating SEs, where general population mortality is assumed to be measured without uncertainty. Bootstrap-based refers to the parametric bootstrap approach used for including uncertainty in population mortality rates in the estimation of SEs. See Table 1 for information on different settings and scenarios. Results are presented for men, aged 55, 65, 75 and 85 years at diagnosis. For setting 1 general population mortality rates are stratified by age, sex and calendar year. For setting 2 general population mortality rates are stratified by age, sex, calendar year and region

**Fig. 2:**
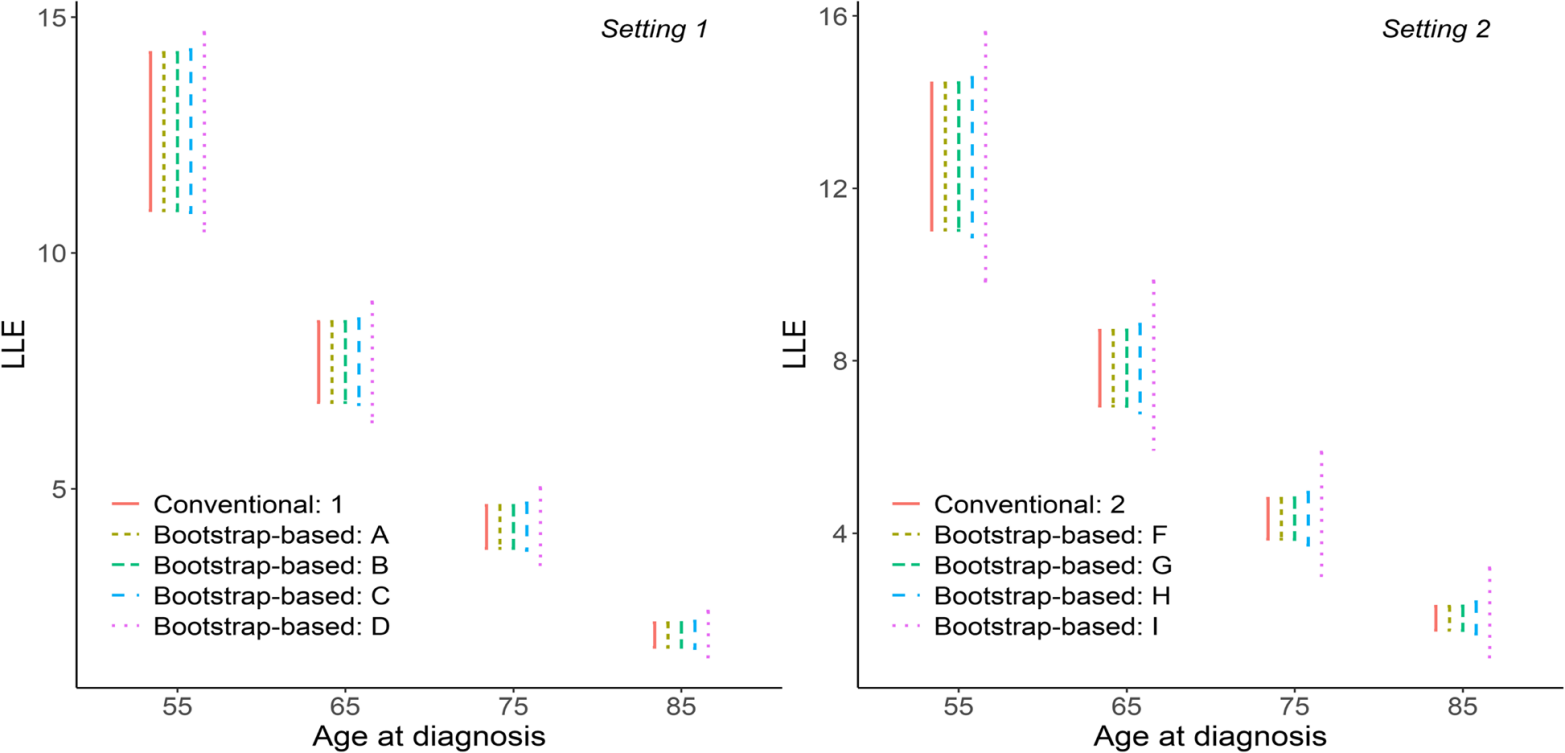
Confidence intervals of LLE. Confidence intervals of loss in life expectancy (LLE) from different methods, settings and scenarios for including uncertainty in the general population mortality when estimating SEs. Conventional refers to the standard method for estimating SEs, where general population mortality is assumed to be measured without uncertainty. Bootstrap-based refers to the parametric bootstrap approach used for including uncertainty in population mortality rates in the estimation of SEs. See Table 1 for information on different settings and scenarios. Results are presented for men, aged 55, 65, 75 and 85 years at diagnosis and LLE estimates for the Stockholm region. For setting 1 general population mortality rates are stratified by age, sex and calendar year. For setting 2 general population mortality rates are stratified by age, sex, calendar year and region. The LLE is measured in years

CIs of 5-year RS and LLE for each of the 10 scenarios A-J, for males and by selected ages at diagnosis (55, 65, 75, 85) are illustrated in Figs. 1-2. Visually, differences in the length of Cis of 5-year RS can be observed only for scenarios D and I. For the length of CIs of LLE differences are seen for scenarios D, I and H.

Graphical comparisons of the bootstrap-based and conventional estimates of 5-year RS, LLE and their CIs from each of the 10 scenarios A-J, for males aged 50+ are found in Additional file 3.

### Marginal estimates

Marginal measures are presented in Table 5 for each of the 10 scenarios from Table 1. We can here observe similar patterns to conditional results presented in Tables 2, 3 and 4. For instance, for scenarios E and J (using the reduced cancer cohort), there is no inflation in SEs of marginal 5-year RS or LLE. Also, changes in SEs of marginal 5-year RS can be seen in scenarios D and I (the general population is 0.05% of the original size), for LLE these changes are observed also for scenarios C and H (the general population is 0.5% of the original size).

**Table 5:** Marginal estimates of 5-year RS and LLE for all investigated settings and scenarios. Marginal point estimates (PE) of 5-year relative survival (RS) and loss in life expectancy (LLE), with lower (LCI) and upper (UCI) confidence intervals, standard errors (SE) and relative % precision (RP) from different methods, settings and scenarios for including uncertainty in the general population mortality when estimating SEs. For settings 1 and 1S general population mortality rates are stratified by age, sex and calendar year. For settings 2 and 2S general population mortality rates are stratified by age, sex, calendar year and region. RP illustrates comparison of the conventional method to a bootstrap-based method

Method	Setting / scenario^b^	5-year Relative Survival (RS)					Loss in life expectancy (LLE)^a^				
		PE	SE	LCI	UCI	RP^c^	PE	SE	LCI	UCI	RP
Conventional^d^	1	0.632	0.011	0.611	0.654	0.00	5.40	0.16	5.09	5.71	0.00
Bootstrap-based^e^	A	0.632	0.011	0.611	0.654	0.01	5.40	0.16	5.09	5.71	0.11
	B	0.632	0.011	0.611	0.654	0.16	5.40	0.16	5.08	5.71	1.08
	C	0.632	0.011	0.610	0.655	3.08	5.40	0.17	5.06	5.74	21.82
	D	0.632	0.013	0.608	0.658	30.55	5.40	0.28	4.85	5.95	214.25
Conventional	2	0.631	0.011	0.610	0.653	0.00	5.40	0.16	5.09	5.71	0.00
Bootstrap-based	F	0.631	0.011	0.610	0.653	0.02	5.40	0.16	5.09	5.71	0.11
	G	0.631	0.011	0.610	0.653	0.15	5.40	0.16	5.09	5.72	1.09
	H	0.631	0.011	0.609	0.654	3.07	5.41	0.18	5.07	5.76	22.41
	I	0.631	0.013	0.606	0.656	30.06	5.58	0.32	4.95	6.21	313.01
Conventional	1S^f^	0.619	0.037	0.550	0.695	0.00	5.19	0.51	4.19	6.19	0.00
Bootstrap-based	E	0.619	0.037	0.550	0.695	0.02	5.19	0.51	4.19	6.19	0.11
Conventional	2S^f^	0.617	0.037	0.549	0.693	0.00	5.19	0.51	4.19	6.19	0.00
Bootstrap-based	J	0.616	0.037	0.549	0.693	-0.01	5.19	0.51	4.19	6.19	0.15

^a^LLE is presented in years

^b^See Table 1 for information on the different settings and scenarios

^c^RP is presented in %

^d^Conventional refers to the standard method for estimating SEs, where the general population mortality is assumed to be measured without uncertainty

^e^Bootstrap-based refers to the parametric bootstrap approach used for including uncertainty in population mortality rates in the estimation of SEs

^f^S stands for small and refers to the setting to investigate what would be observed for a smaller population

## Discussion

In this study we found that when the whole general population, i.e. all people living in a country or region, that is the catchment area for the population-based cancer registry, is used to get predicted mortality rates for estimating 5-year RS or the LLE, the assumption of known (fixed) general population mortality rates has a negligible effect on the estimates. The relative precision for both 5-year RS and LLE was less than 1%. This is an important message for population based cancer research.

The impact of including the uncertainty in expected mortality was larger when the population mortality was stratified on more variables, here region. However, the impact was still small when the mortality rates were based on the whole population. The largest relative precision for 5-year RS was 0.03% and for the LLE it was 0.46%. Interestingly, it did not make a large difference when we assumed that the cancer cohort was only 10% of the original size and the corresponding reduced general population was used, as would be the case in a smaller country or a region. The relative precision in this case for 5-year RS was in the range of 0.01% to 0.03%, and less than 1% for LLE. This suggests that as long as the whole general population is used, regardless of the size of a country or region a possible variation in the expected mortality rates can be ignored.

If the whole population of the country or region is not available, then the validity of the assumption of known expected mortality rates should be discussed. In the study we illustrated that for 5-year RS when the general population was reduced to 0.05% of the original size and stratified by age, sex and calendar year, the relative precision was 15 (7)% and 40 (34)% for males (females) 75 and 85 years old, respectively. The LLE was affected to a larger extent than RS. For all ages the increase in SE of the LLE was observed when the general population was reduced to 0.5% and 0.05% of the original size. The relative precision for the LLE when the general population was reduced to 0.05% and stratified by age, sex and calendar year was 221 (129)% and 245 (176)% for males (females) 75 and 85 years old, respectively. For estimates in older ages the impact was larger possibly because the expected mortality rates of elderly patients in the general population are more influential than for younger patients and, therefore, the uncertainty introduced in the general mortality could have a larger impact on SEs of both 5-year RS and LLE. For marginal 5-year RS the relative precision was 3% when the general population was reduced to 0.5% of the original size and 30% when reduced to 0.05% of the original size. Similar to above-described conditional estimates, the marginal estimates of the LLE showed larger relative precision than the marginal 5-year RS with 22% and 313% for the general population reduced to 0.5% and 0.05% of the original size, respectively.

Previous work in this area has focused on non-parametric estimates of RS, and the results were similar to our results [5]. Another study did not address the uncertainty in the general population mortality rates, but investigated the impact on SE of non-parametric estimates of RS when allowing the expected survival for the cancer cohort to vary [20]. Non-parametric bootstrap was used to sample from the cancer cohort, resulting in a different age and sex distribution in each sample. Hence, the expected survival calculated for the non-parametric estimate of RS varied in each sample. However, it was still assumed that the general population mortality used to obtain this expected survival was fixed.

Even though the results of our study suggest that the assumption of known expected mortality rates is reasonable when based on the whole population, we did not investigate all possible situations. There might be situations when the general population mortality rates are stratified on even more covariates, leading to very small groups. Another aspect we did not include is the situation when more covariates are included in both the excess and expected mortality. We assumed that region did not have an impact on excess mortality, even when region was included for the expected mortality. Also, it would be of interest to elaborate on the findings using data on other cancer types. In addition, we used a modelling approach to obtain smooth estimates of the general population mortality rates, instead of using the raw numbers of deaths and person-years in each strata. An alternative way to create varying population mortality rates could be bootstraping from raw numbers of the number of deaths and person-years.

In conclusion, this study contributes to population-based cancer studies suggesting that in general SE of RS and the LLE give reliable estimates with assumption of *known* expected mortality rates. However, when the general population mortality rates are not based on the whole population, the uncertainty in the estimates of the expected measures should be taken into account as the conventional estimates of SE for relative survival proportions and loss in life expectancy may be too low, particularly for marginal values.

## Supplementary Information

**Additional file 1** Estimates of 5-year RS and LLE for setting 2 and scenarios F-H for the Gotland region.

**Additional file 2** Estimates of 5-year RS and LLE for setting 2 and scenario J for the Gotland region.

**Additional file 3** Graphical comparisons of the bootstrap-based and conventional estimates of 5-year RS, LLE and their CIs for all scenarios.

## Data Availability

The data used for this study may not, according to the ethical permission granted for its use, be shared by the authors to a third party. It is accessible by application to the Swedish authorities (The Swedish Cancer Registry).

## Abbreviations

RS: relative survival
LLE: loss in life expectancy
SE: standard error
FPM: flexible parametric survival model
CI: confidence interval
df: degree of freedom
RP: the relative % precision
PE: point estimate
