# Supplementary material for: Assessing the impact of including variation in general population mortality on standard errors of relative survival and loss in life expectancy
Source: BMC Med Res Methodol. 2022 May 2;22:130. doi: 10.1186/s12874-022-01597-7 (PMC9059421; doi:10.1186/s12874-022-01597-7)
Supplement: Supplementary file 1 — Additional file 1 Estimates of 5-year RS and LLE for setting 2 and scenarios F-H for the Gotland region. [file 12874_2022_1597_MOESM1_ESM.pdf]

Method	Setting / scenario[b]	5-year Relative Survival (RS) PE	SE	LCI	UCI	RP[c]	Loss in life expectancy (LLE)[a,f] PE	SE	LCI	UCI	RP
men, 55 years at diagnosis											
Conventional [d]	2	0.639	0.026	0.585	0.687	0.00	12.77	0.87	11.06	14.48	0.00
Bootstrap[e]	F	0.639	0.026	0.585	0.687	0.00	12.77	0.88	11.05	14.49	1.56
-based	G	0.639	0.026	0.585	0.687	0.01	12.77	0.93	10.94	14.60	14.71
	H	0.639	0.026	0.585	0.687	0.23	12.71	1.83	9.13	16.29	339.17
men, 65 years at diagnosis											
Conventional	2	0.636	0.021	0.593	0.675	0.00	7.78	0.44	6.91	8.65	0.00
Bootstrap	F	0.636	0.021	0.593	0.675	0.00	7.78	0.45	6.90	8.66	3.46
-based	G	0.636	0.021	0.593	0.675	0.03	7.79	0.51	6.79	8.79	33.11
	H	0.636	0.021	0.593	0.676	0.55	7.81	1.33	5.21	10.40	797.79
men, 75 years at diagnosis											
Conventional	2	0.629	0.021	0.586	0.669	0.00	4.19	0.23	3.73	4.65	0.00
Bootstrap	F	0.629	0.021	0.586	0.669	0.01	4.19	0.24	3.72	4.66	6.31
-based	G	0.629	0.021	0.586	0.669	0.07	4.20	0.30	3.62	4.79	64.14
	H	0.629	0.021	0.586	0.670	1.43	4.30	1.01	2.31	6.28	1790.86
men, 85 years at diagnosis											
Conventional	2	0.581	0.030	0.520	0.638	0.00	1.92	0.13	1.66	2.17	0.00
Bootstrap	F	0.581	0.030	0.520	0.638	0.03	1.92	0.14	1.65	2.18	6.72
-based	G	0.581	0.030	0.519	0.638	0.20	1.93	0.17	1.59	2.27	72.67
	H	0.580	0.031	0.517	0.638	3.95	2.04	0.72	0.63	3.45	2852.80
women, 55 years at diagnosis											
Conventional	2	0.662	0.025	0.611	0.709	0.00	12.15	0.91	10.37	13.92	0.00
Bootstrap	F	0.662	0.025	0.611	0.709	0.00	12.14	0.91	10.36	13.93	0.70
-based	G	0.662	0.025	0.611	0.709	0.01	12.14	0.94	10.29	13.99	8.49
	H	0.663	0.025	0.611	0.709	0.36	12.08	1.54	9.06	15.09	188.28
women, 65 years at diagnosis											
Conventional	2	0.660	0.020	0.619	0.697	0.00	7.78	0.47	6.86	8.70	0.00
Bootstrap	F	0.660	0.020	0.619	0.697	0.01	7.78	0.47	6.85	8.71	1.70
-based	G	0.660	0.020	0.619	0.697	0.02	7.78	0.51	6.77	8.79	19.81
	H	0.660	0.020	0.619	0.697	0.48	7.79	1.09	5.65	9.92	436.59
women, 75 years at diagnosis											
Conventional	2	0.653	0.020	0.612	0.692	0.00	4.52	0.27	3.99	5.05	0.00
Bootstrap	F	0.653	0.020	0.612	0.692	0.01	4.52	0.27	3.98	5.05	3.08
-based	G	0.653	0.020	0.612	0.692	0.03	4.52	0.31	3.91	5.13	35.67
	H	0.654	0.020	0.612	0.692	0.66	4.60	0.84	2.96	6.24	870.51
women, 85 years at diagnosis											
Conventional	2	0.607	0.027	0.553	0.658	0.00	2.20	0.15	1.91	2.49	0.00
Bootstrap	F	0.608	0.027	0.553	0.658	0.03	2.20	0.15	1.90	2.50	3.55
-based	G	0.607	0.027	0.552	0.658	0.15	2.20	0.18	1.85	2.55	40.88
	H	0.607	0.027	0.551	0.658	3.13	2.30	0.54	1.24	3.36	1215.10

[a] LLE is presented in years.
[b] See Table 1 for information on the different settings and scenarios.
[c] RP is presented in %.
[d] Conventional refers to the standard method for estimating SEs, where the general population mortality is assumed to be measured without uncertainty.
[e] Bootstrap-based refers to the parametric bootstrap approach used for including uncertainty in observed population mortality rates in the estimation of SEs.
[f] LLE estimates are presented for the Gotland region

Table S1: Point estimates (PE) of 5-year relative survival (RS) and loss in life expectancy (LLE), with lower (LCI) and upper (UCI) confidence intervals, standard errors (SE) and relative % precision (RP) from setting 2, different methods and scenarios for including uncertainty in the general population mortality when estimating SEs. Results are presented for men and women, aged 55, 65, 75 and 85 years at diagnosis and LLE estimates are for the Gotland region. General population mortality rates are stratified by age, sex, calendar year and region. RP illustrates comparison of the conventional method to a bootstrap-based method.
