# Supplementary material for: Assessing the impact of including variation in general population mortality on standard errors of relative survival and loss in life expectancy
Source: BMC Med Res Methodol. 2022 May 2;22:130. doi: 10.1186/s12874-022-01597-7 (PMC9059421; doi:10.1186/s12874-022-01597-7)
Supplement: Supplementary file 3 — Additional file 3 Graphical comparisons of the bootstrap-based and conventional estimates of 5-year RS, LLE and their CIs for all scenarios. [file 12874_2022_1597_MOESM3_ESM.pdf]

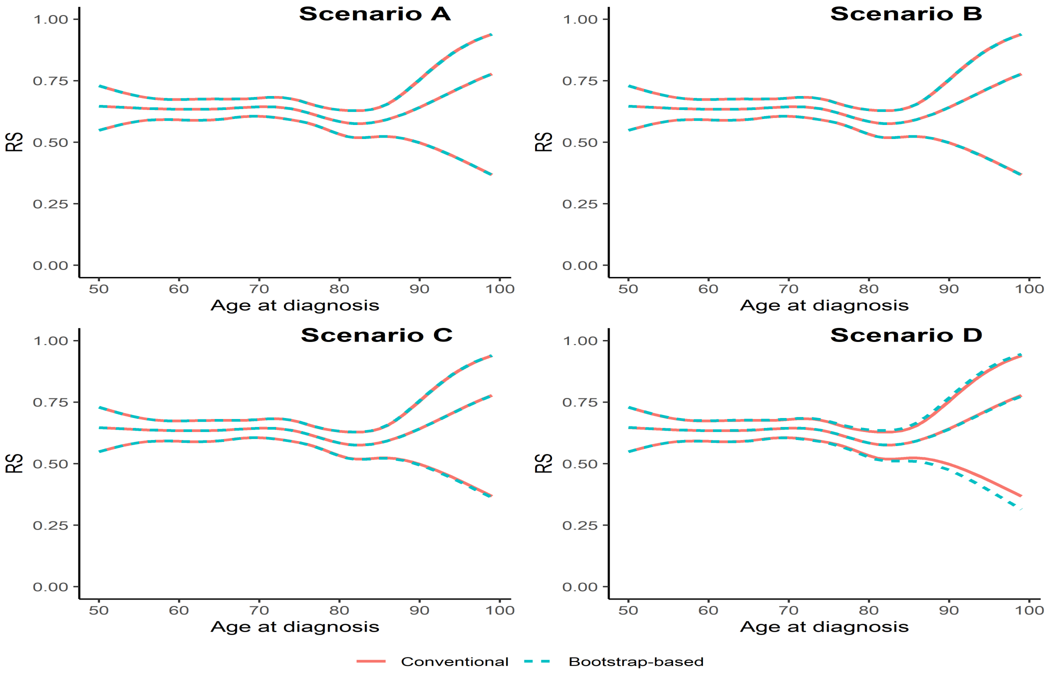

(a) 5-year Relative Survival (RS) (setting 1)

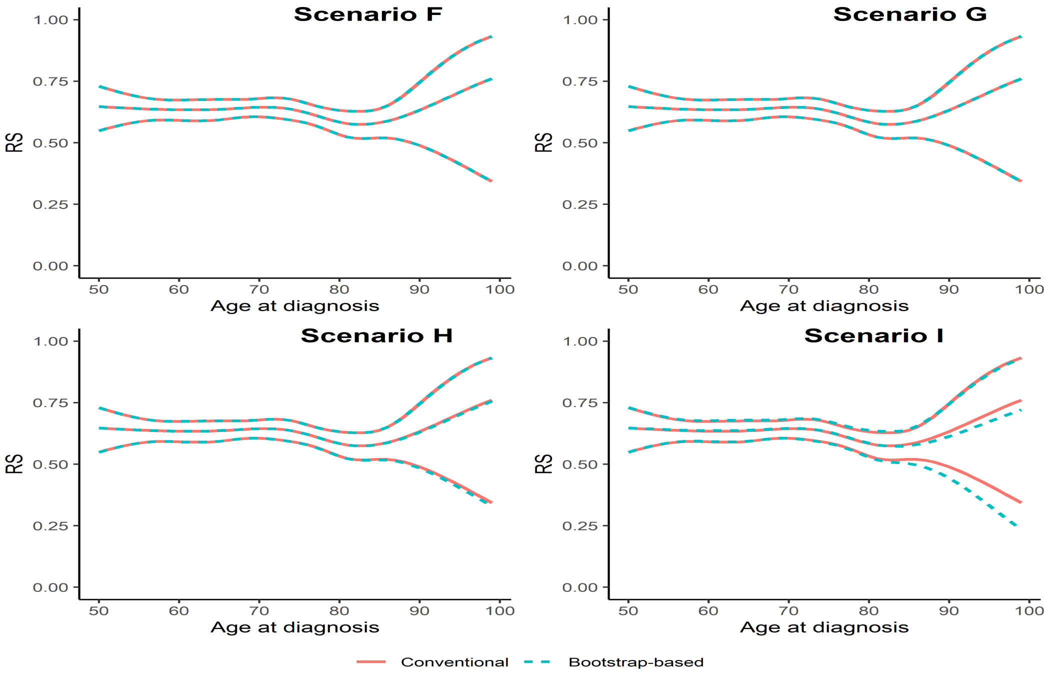

(b) 5-year Relative Survival (RS) (setting 2)

Figure S1: Point estimates of 5-year relative survival (RS) with lower and upper confidence intervals from different methods, settings and scenarios for including uncertainty in the general population mortality when estimating SEs. Conventional refers to the standard method for estimating SEs, where general population mortality is assumed to be measured without uncertainty. Bootstrap-based refers to the parametric bootstrap approach used for including uncertainty in population mortality rates in the estimation of SEs. See Table 1 for information on different settings and scenarios. Results are presented for men aged 50+ years at diagnosis. For setting 1 general population mortality rates are stratified by age, sex and calendar year. For setting 2 general population mortality rates are stratified by age, sex, calendar year and region

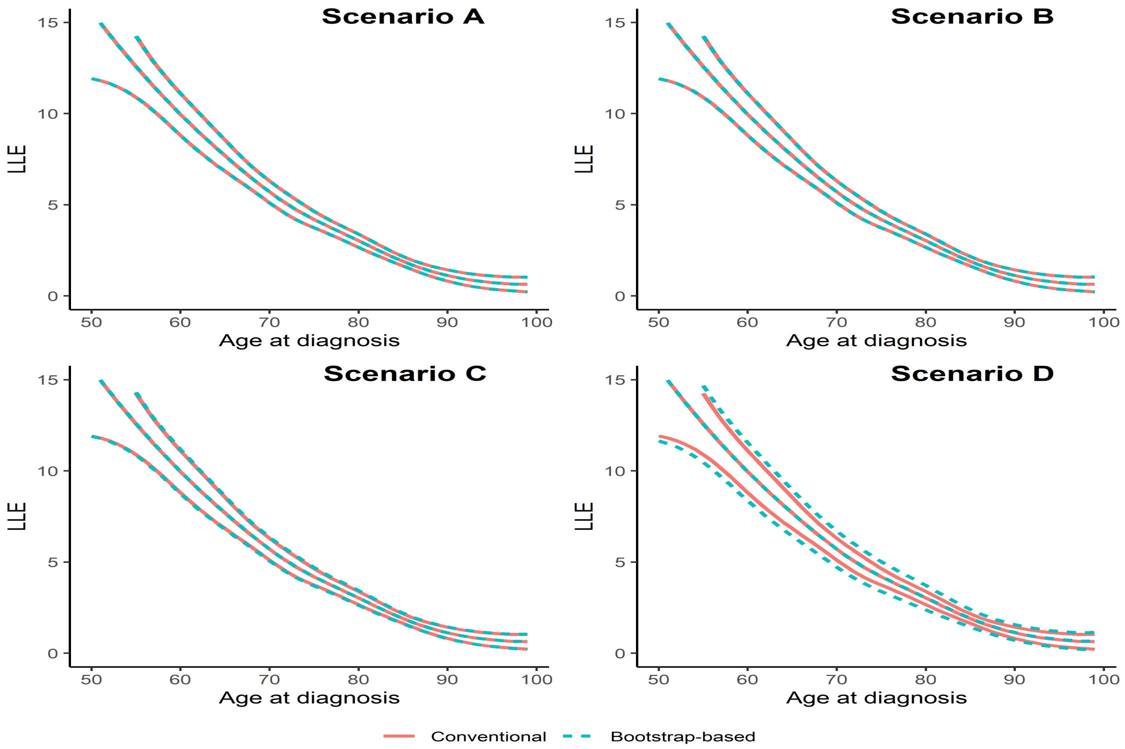

(a) Loss in life expectancy (LLE) (setting 1)

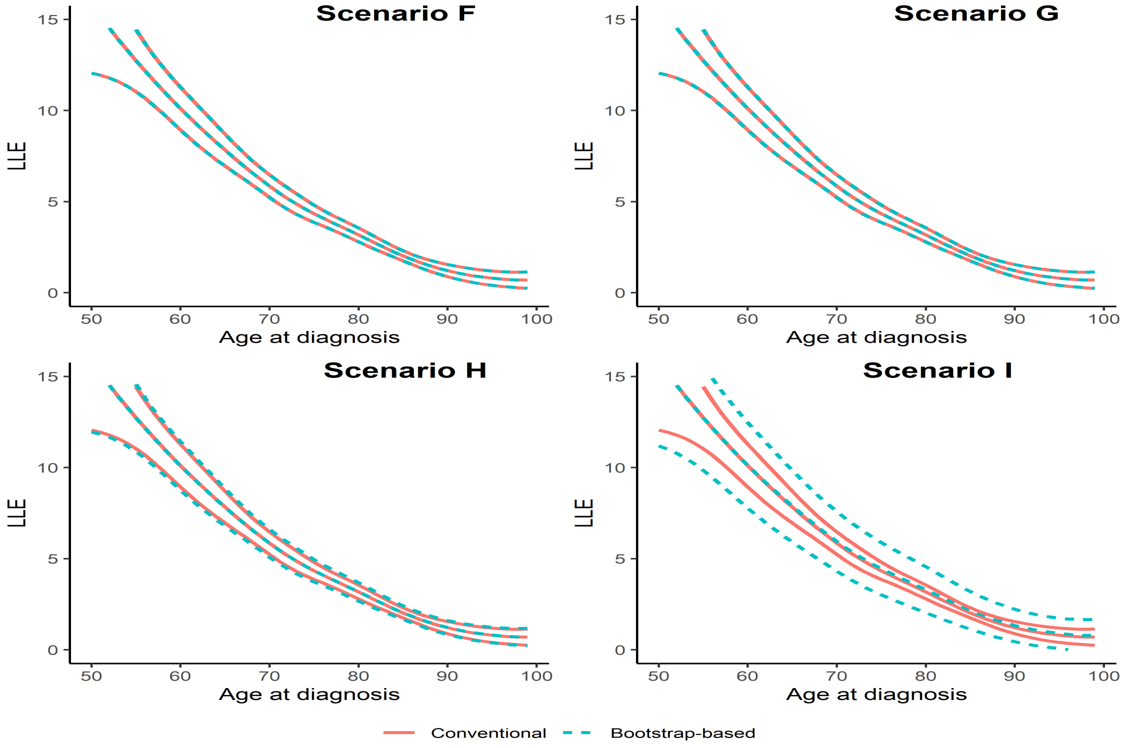

(b) Loss in life expectancy (LLE) (setting 2)

Figure S2: Point estimates of loss in life expectancy (LLE) with lower and upper confidence intervals from different methods, settings and scenarios for including uncertainty in the general population mortality when estimating SEs. Conventional refers to the standard method for estimating SEs, where general population mortality is assumed to be measured without uncertainty. Bootstrap-based refers to the parametric bootstrap approach used for including uncertainty in population mortality rates in the estimation of SEs. See Table 1 for information on different settings and scenarios. Results are presented for men aged 50+ years at diagnosis and in setting 2 the LLE estimates are for the Stockholm region. For setting 1 general population mortality rates are stratified by age, sex and calendar year. For setting 2 general population mortality rates are stratified by age, sex, calendar year and region. The LLE is measured in years

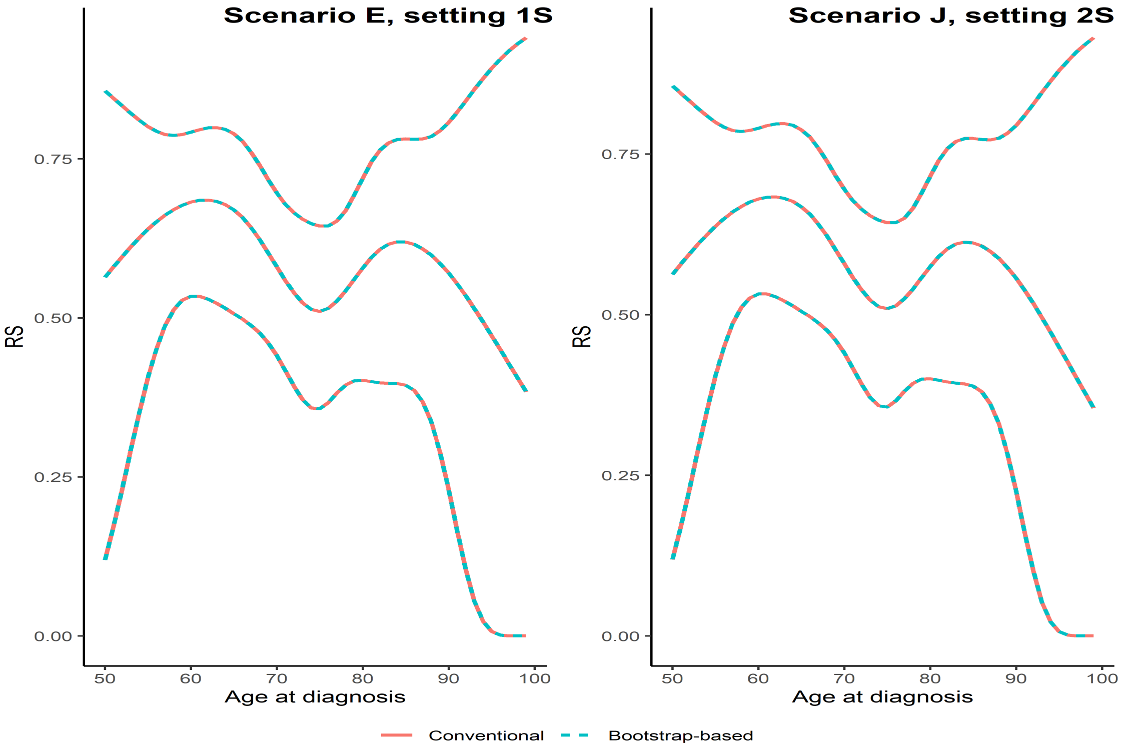

(a) 5-year Relative Survival (RS)

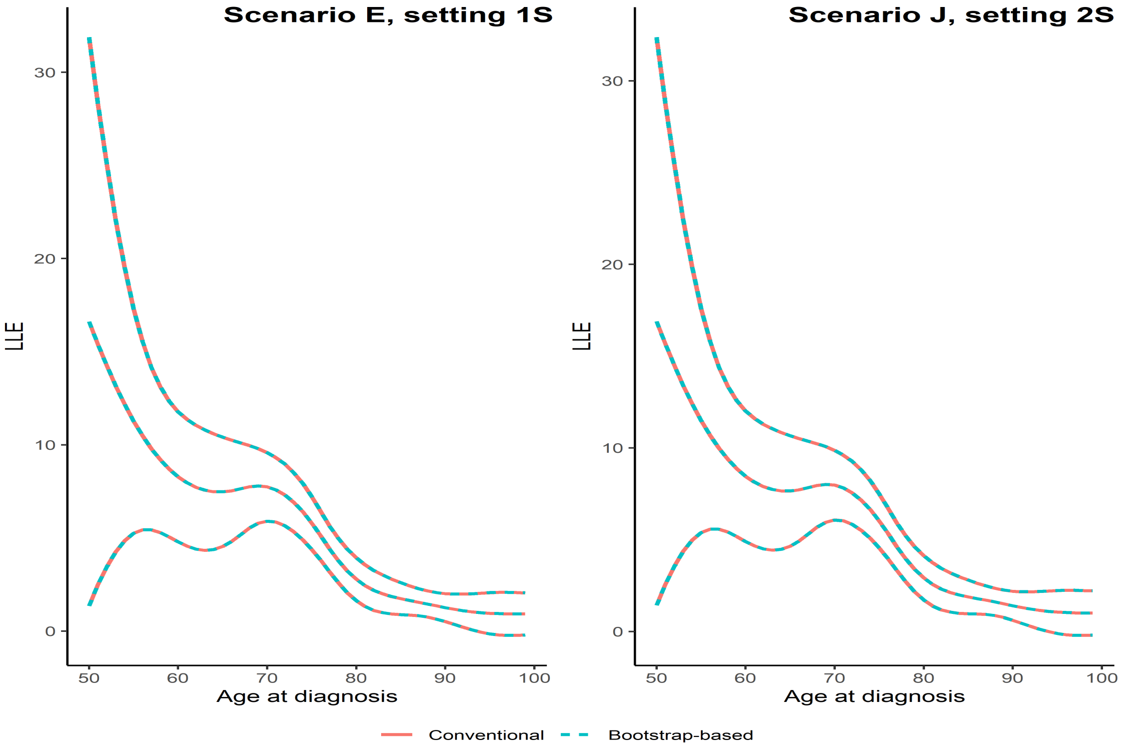

(b) Loss in life expectancy (LLE)

Figure S3: Point estimates of 5-year relative survival (RS) and loss in life expectancy (LLE) with lower and upper confidence intervals from different methods, settings and scenarios for including uncertainty in the general population mortality when estimating SEs. Conventional refers to the standard method for estimating SEs, where general population mortality is assumed to be measured without uncertainty. Bootstrap-based refers to the parametric bootstrap approach used for including uncertainty in population mortality rates in the estimation of SEs. See Table 1 for information on different settings and scenarios. Results are presented for men aged 50+ years at diagnosis and in setting 2S the LLE estimates are for the Stockholm region. For setting 1S general population mortality rates are stratified by age, sex and calendar year. For setting 2S general population mortality rates are stratified by age, sex, calendar year and region. Here S stands for small and refers to the setting to investigate what would be observed for a smaller population. The LLE is measured in years
